# THAP9-AS1/miR-133b/SOX4 positive feedback loop facilitates the progression of esophageal squamous cell carcinoma

**DOI:** 10.1038/s41419-021-03690-z

**Published:** 2021-04-14

**Authors:** Jiwei Cheng, Haibo Ma, Ming Yan, Wenqun Xing

**Affiliations:** Department of Thoracic Surgery, The Affiliated Cancer Hospital of Zhengzhou University, Henan Cancer Hospital, 450008 Zhengzhou, China

**Keywords:** Cancer, Cell signalling, Non-coding RNAs

## Abstract

Esophageal squamous cell carcinoma (ESCC) is one of the most common malignant tumors in the digestive system with a high incidence and poor prognosis. Long non-coding RNAs (LncRNA) have been reported to be closely associated with the occurrence and development of various human cancers. Data from GSE89102 shows an increase of THAP9-AS1 expression in ESCC. However, its functions and mechanisms underlying ESCC progression remain to be investigated. In this study, we found that THAP9-AS1 was overexpressed in ESCC tissues and cells. High THAP9-AS1 expression was positively correlated with tumor size, TNM stage, lymph node metastasis, and worse prognosis. Functionally, depletion of THAP9-AS1 suppressed cell proliferation, migration, and invasion, while enhanced apoptosis in vitro. Consistently, knockdown of THAP9-AS1 inhibited xenograft tumor growth in vivo. Mechanistically, THAP9-AS1 could serve as a competing endogenous RNA (ceRNA) for miR-133b, resulting in the upregulation of SOX4. Reciprocally, SOX4 bound to the promoter region of THAP9-AS1 to activate its transcription. Moreover, the anti-tumor property induced by THAP9-AS1 knockdown was significantly impaired due to miR-133b downregulation or SOX4 overexpression. Taken together, our study reveals a positive feedback loop of THAP9-AS1/miR-133b/SOX4 to facilitate ESCC progression, providing a potential molecular target to fight against ESCC.

## Introduction

As one of the most prevalent malignant tumors in the digestive system, esophageal carcinoma ranks ninth in cancer morbidity and is the six-leading cause of cancer-related death globally^1^. Esophageal squamous cell carcinoma (ESCC) represents the major pathological type of esophageal carcinoma outside of the United States, occupying nearly 90% of all cases worldwide^2^. In addition to low socioeconomic status, cigarette smoking, alcohol use, hot beverages drinking, nitrosamines exposure, and micronutrient deficiencies are also regarded to be responsible for the development of ESCC^3^. In China, ESCC accounts for about half of all ESCC cases on earth, with a high incidence in the area of Taihang Mountains, including Hebei, Shanxi, and Henan Provinces^4^. ESCC is characterized by a high mortality rate and poor prognosis because patients are always asymptomatic until the very late stages of the disease. In recent years, cytotoxic agents, molecular-targeting agents, and immunotherapeutic agents have been applied as a systemic treatment for advanced ESCC^5^. Nevertheless, the 5-year relative survival rate for esophageal carcinoma patients at the distant stage is merely 5%^6^. In an attempt to improve the clinical outcomes of this devastating disease, exploring the possible molecular mechanisms behind ESCC progression is imperative.

Long non-coding RNAs (LncRNAs) are a subgroup of transcripts comprising of over 200 nucleotides in size and have no ability to encode protein. LncRNAs are capable of tuning gene expression and affecting cellular signaling cascades through chromatin remodeling, as well as transcriptional and post-transcriptional regulation^7^. A multitude of evidence identifies lncRNAs as tumor suppressors or drivers via influencing various cellular processes important for normal development and physiology, highlighting their great potential in cancer diagnosis, prognosis, and therapy^8,9^. There is a growing list of researches concerning the involvement of lncRNAs in ESCC initiation and progression^10^. For instance, Wang et al. reported that LOC440173 facilitated ESCC cell proliferation, migration, invasion, and epithelial-mesenchymal transition (EMT) process in vitro, and promoted tumor growth in vivo via competitively sponging miR-30d-5p to upregulate histone deacetylase 9 (HDX9) expression^11^. Liang et al. demonstrated that CASC9 interacted with CREB-binding protein (CBP) to increase LAMC2 expression, thereby stimulating FAK-PI3K/Akt signaling pathways to accelerate ESCC metastasis^12^. However, the studies of lncRNAs in the ESCC context remain in their infancy. LncRNA-dependent gene regulation mechanisms in ESCC deserve in-depth exploration in order to develop personalized therapeutic targets.

THAP9 antisense RNA 1 (THAP9-AS1), a lncRNA located on 4q21.22, was previously discovered to be upregulated in nasopharyngeal carcinoma and breast cancer by using next-generation deep sequencing^13,14^. Moreover, THAP9-AS1 was delineated as an oncogenic factor in pancreatic ductal adenocarcinoma (PDAC)^15^ and gastric cancer^16^. According to a publicly available GEO database (GSE89102), we found that THAP9-AS1 was highly expressed in 5 ESCC tumor tissues compared with adjacent non-cancerous tissues. However, its precise biological function and pathological mechanism in ESCC progression remain undefined.

MiRNAs, short RNA molecules with 19 to 25 nucleotides in length, are able to modulate physiological processes and cancer pathogenesis by inducing mRNA degradation or translational inhibition^17^. A generalized action mechanism for lncRNAs is serving as endogenous miRNA sponges to sequester them from binding to the 3′-UTR of target protein-coding genes^18^. Hence, this study intends to unearth whether THAP9-AS1 exerts regulatory effects in ESCC according to the similar “lncRNA-miRNA-mRNA” mechanism.

In this study, THAP9-AS1 was found to be upregulated in ESCC and predicted a worse prognosis. Moreover, THAP9-AS1 was positively correlated with tumor size, TNM stage, and lymph node metastasis. Functionally, silencing of THAP9-AS1 suppressed ESCC cell proliferation, migration, and invasion in vitro and reduced tumor growth in vivo. Mechanistically, THAP9-AS1 acted as a molecular sponge of miR-133b to promote the expression of its target SOX4. Interestingly, SOX4 could bind to the promoter of THAP9-AS1 to activate its transcription. In summary, we proposed a positive feedback loop of THAP9-AS1/miR-133b/SOX4 in the process of ESCC, providing a novel prognostic biomarker and therapeutic target for ESCC patients.

## Materials and methods

### Clinical tissue specimens

A cohort of 68 ESCC patients who had received radical resection at the Affiliated Cancer Hospital of Zhengzhou University from March 2016 to February 2018 was recruited to enroll in this study. There was no adjuvant anticancer therapy for all patients prior to surgery. All harvested tissues were confirmed by pathological means by pathologists who were blinded to the clinical data. The main clinicopathological characteristics of ESCC cases were summarized in Supplementary Table S1. Once collected, tumor tissue samples and adjacent normal tissues (at least 5 cm from the primary lesion) were instantly frozen in liquid nitrogen and maintained at −80 °C. The experimental procedures were executed under the approval of the Ethics Review Committee of the Affiliated Cancer Hospital of Zhengzhou University and strictly conformed to the Declaration of Helsinki. Informed written consent was obtained from each participant before the usage of their tissues.

### Cell culture

Human ESCC cell lines (KYSE-150, YES2, TE-1, Eca-109, and KYSE-30) and normal immortalized esophageal epithelial cell line Het-1A were obtained from American Type Culture Collection (ATCC; Manassas, VA, USA). All cells were maintained in RPMI 1640 medium (Gibco, Rockville, MD, USA) plus 10% fetal bovine serum and 1% penicillin/streptomycin in a humidified atmosphere containing 5% CO_2_ at 37 °C. Cell passage was routinely performed when cell confluence reached approximately 80%. Short tandem repeat (STR) fragment analysis was used to perform cell line authentication and all cell lines were free of mycoplasma contamination.

### Cell transfection

Small interfering RNAs (siRNAs) against THAP9-AS1 (si-THAP9-AS1#1, si-THAP9-AS1#2), siRNA targeting SOX4 (si-SOX4), negative control non-targeting siRNA (si-NC), miR-133b mimic/inhibitor (miR-133b/anti-miR-133b), and mimic control (miR-NC) were purchased from GenePharma (Shanghai, China). THAP9-AS1-overexpressing or SOX4-overexpressing plasmid was constructed by Geneseed (Guangzhou, China). When cell confluence reached 60%, 40 nM of oligonucleotides or 5 μg of plasmids were transfected into ESCC cells by using Lipofectamine 2000 kit (Invitrogen, Carlsbad, CA, USA) following the manufacturer’s manual.

### Quantitative real-time polymerase chain reaction (qRT-PCR)

Total RNA was extracted from clinical tissues or cells using TRIzol Reagent (Sigma-Aldrich, St Louis, MO, USA). For THAP9-AS1 and SOX4, total RNA was reversely transcribed into cDNA with a Transcriptor First Strand cDNA Synthesis Kit (Roche Diagnostics, Penzberg, Germany). For miR-133b, the first-strand cDNA was synthesized from total RNA by using miRNA 1st Strand cDNA Synthesis Kit (Vazyme, Nanjing, China). Quantitative RT-PCR reaction was conducted by using TaqMan Gene Expression Master Mix (Applied Biosystems, Foster City, CA, USA) on an ABI StepOne Real-time PCR system (Applied Biosystem). Gene expression changes were calculated by the 2^−ΔΔCT^ method. Glyceraldehyde-3-phosphate dehydrogenase (GAPDH) or U6 snRNA was applied as the internal reference to normalize the target gene expression. The primer sequences used are described in Supplementary Table S2.

### Cell proliferation assay

The proliferative ability of ESCC cells was evaluated by cell counting kit-8 (CCK-8) and colony-forming assays. For the CCK-8 assay, cells at the logarithmic phase were seeded into 96-well plates at a density of 2 × 10^3^ cells/well. At 1, 2, 3, and 4 days after inoculation, 10 µl of CCK-8 reagent (Biotool, Houston, TX, USA) was added to each well and cultured at 37 °C for 4 h. The absorbance value was read at 450 nm by using a microplate reader Synergy HT (BioTek Instruments, Winooski, VT, USA). As for clonogenic assay, 500 cells were inoculated in 6-well plates and cultured over a span of two weeks to allow for colony formation. In the culture process, the medium was replaced every third day. Then, cells were washed with PBS, fixed with 4% paraformaldehyde, and stained with 0.1% crystal violet. The number of colonies with more than 50 cells was manually counted under a microscope.

### Flow cytometry assay

Annexin V-FITC Apoptosis Detection Kit (Beyotime, Shanghai, China) was applied to measure the apoptotic rate of ESCC cells. Briefly, cells (5 × 10^4^/well) were inoculated in 6-well plates and grew for 48 h. Then, cells were collected, centrifuged at 1000 × *g* for 5 min, and suspended in 195 μl Annexin V-FITC binding buffer. After stained with 5 μl of Annexin-V-FITC labeling solution and 15 μl of PI solution for 20 min at room temperature in the dark, cells were analyzed by a FACSCalibur flow cytometer (BD Biosciences, San Jose, CA, USA) to detect apoptotic rate.

### Wound healing assay

ESCC cells were placed in 6-well plates to form a single confluent cell layer. After removing the culture medium, a sterile 200 μl pipette tip was used to scratch a linear wound across the well center. At 0 h and 48 h after scratch, an inverted microscope was used to capture the images to calculate the rate of wound closure.

### Transwell assay

For migration assays, 5 × 10^4^ ESCC cells suspended in serum-free medium were added to the apical chamber of the Transwell chamber (Costar, Corning Inc., NY, USA), while a medium containing 10% FBS was used to fill the lower compartment. After 24 h of incubation, a cotton swab was utilized to wipe off the residual cells on the upper surface of the inner chamber. Meanwhile, the cells on the other side of the membrane were fixed with methanol and stained with crystal violet. Finally, four random visual fields were taken by a microscope to count the migration cells. For the invasion assay, the same procedures were conducted as described above except that the upper insert was pre-coated with 50 µl Matrigel matrix and the cell number was 1 × 10^5^.

### Western blot assay

Total protein was extracted from cultured cells and tumor tissues by using RIPA lysis and extraction buffer (Thermo Fisher Scientific, Waltham, MA, USA). Protein concentration was measured with a bicinchoninic acid protein assay kit (Thermo Fisher Scientific). An equal amount of protein samples (30 μg) was loaded on 10% sodium dodecyl sulfate-polyacrylamide gel electrophoresis (SDS-PAGE) gel and transferred onto polyvinylidene fluoride (PVDF) membrane (Millipore, Billerica, MA, USA). After being blocked in 5% skim milk for 1 h, the membrane was cultured with primary antibodies against SOX4 (Santa Cruz Biotechnology, Dallas, TX, USA; cat. #sc-130633), Ki-67 (Santa Cruz Biotechnology, cat. #sc-165994), PCNA (Santa Cruz Biotechnology, cat. #sc-9857) or GAPDH (Santa Cruz Biotechnology, cat. #sc-25778) overnight at 4 °C and subsequently with goat anti-mouse IgG-HRP (Santa Cruz Biotechnology, cat. #sc-2060) at room temperature for 2 h. The immunoreactive bands were visualized by using Amersham ECL Prime Western blotting reagent (GE Healthcare Life Sciences, Uppsala, Sweden) and quantified by Image Lab Software Version 5.2.1 (Bio-Rad Laboratories Inc., Hercules, California, USA).

### Nuclear-cytoplasmic fractionation

Nuclear and cytoplasmic fractions separation of ESCC cells was performed by using a PARIS kit protein and RNA isolation system (Life Technologies, Carlsbad, CA, USA) according to the manufacturer’s guidance. The expression patterns of THAP9-AS1, GAPDH, and U6 in different fractions were determined by qRT-PCR. GAPDH and U6 were used as respective control for cytoplasmic RNA and nuclear RNA.

### Bioinformatics prediction

ESCC chip data (GSE89102, GSE100942, GSE23400, GSE26886, GSE17351, GSE44021) and ESCC miRNA expression profiling database GSE43732 were obtained from the GEO database (www.ncbi.nlm.nih.gov/geo) to investigate the differentially expressed genes in ESCC tumor tissues and adjacent non-cancerous tissues. DIANA-LncBase (http://carolina.imis.athena-innovation.gr/diana_tools/web/index.php?r=lncbasev2%2Findex) and LncBook (https://bigd.big.ac.cn/lncbook/index) were applied to predict the potential miRNAs that could interact with THAP9-AS1. TargetScan (http://www.targetscan.org/vert_72/), miRDB (http://mirdb.org/), DIANA-microT-CDS (http://diana.imis.athena-innovation.gr/DianaTools/index.php?r=microT_CDS/index) and starBase v3.0 (http://starbase.sysu.edu.cn) were used to predict the candidate targets of miR-133b. Web-based tool GEPIA (http://gepia.cancer-pku.cn/detail.php?gene=&clicktag=boxplot) was used to analyze the expression of THAP9-AS1 and SOX4 in tumor tissues and normal tissues of esophageal carcinoma.

### Plasmid construction and dual-luciferase reporter assay

The wild-type sequences of THAP-AS1 containing miR-133b target sites were inserted to the downstream of the Renilla luciferase (hRluc) gene in the psiCHECK-2 expression vector (Promega, Madison, WI, USA), named as THAP9-AS1-wt1 or THAP9-AS1-wt2. The corresponding mutant luciferase reporter vectors (THAP9-AS1-mut1 or THAP9-AS1-mut2) were constructed by replacing nucleotides in THAP9-AS1 that were complementary to miR-133b by using QuikChange XL Site-Directed Mutagenesis kit (Agilent Technologies, Santa Clara, CA, USA). ESCC cells were transfected with 50 ng of the constructed vector and 20 nM miR-133b or miR-NC by using Lipofectamine 2000 (Invitrogen, Carlsbad, CA, USA). Firefly and Renilla luciferase activities were measured using a Dual-Glo Luciferase assay kit (Promega) following the manufacturer’s recommendations. The firefly reporter gene served as the reference gene to normalize the activity of Renilla luciferase. For examining the binding between SOX4 and miR-133b, SOX4-wt or SOX4-mut luciferase reporter were built to perform similar procedures.

### RNA immunoprecipitation (RIP) assay

EZMagna RIP kit (Millipore, Billerica, MA, USA) was utilized to perform RIP experiments. In brief, miR-NC-transfected or miR-133b-transfected ESCC cells were lysed with the RIP lysis buffer. Then, cell lysates were incubated with RIP buffer containing magnetic beads conjugated with specific antibodies against Ago2 or negative control IgG overnight at 4 °C with rotation. After being incubated with Proteinase K, the qRT-PCR assay was used to measure the expression of THAP9-AS1 in coprecipitated RNA.

### Biotin-coupled miRNA pull-down assay

The biotinylated miRNA mimic (Bio-miR-133b, GenePharma, Shanghai, China) and negative control (Bio-NC) transfection was performed when ESCC cells reach approximately 60% confluence. After 48 h, cells were collected, washed with PBS, and lysed in the lysis buffer. Then, cell lysates were incubated with streptavidin-coated magnetic beads at 4 °C for 2 h to pull down the biotin-coupled RNA complex. qRT-PCR analysis was used to detect the enrichment of SOX4 mRNA in the bound fraction.

### Chromatin immunoprecipitation (ChIP) assay

To determine the binding of SOX4 on the promoter of THAP9-AS1, a ChIP assay was applied by using an EZ-ChIP Chromatin immunoprecipitation kit (Millipore, Bedford, MA, USA) in accordance with the manufacturer’s protocol. Briefly, ESCC cells were incubated with 1% formaldehyde for 10 min to form DNA-protein cross-linking. Then, cells were lysed in lysis buffer and sonicated to generate chromatin fragments ranging from 200 to 1000 bp. Next, the chromatin was immunoprecipitated with magnetic protein A beads coupled with SOX4 antibody or control IgG. the qRT-PCR assay was conducted to quantify the precipitated DNA.

### Xenograft mouse model

A total of 10 male BALB/c nude mice (age, 4–5 weeks; weight, 18–22 g) were purchased from Beijing Vital River Laboratory Animal Technology Co. Ltd. (Beijing, China) and fed under a specific pathogen-free (SPF) environment. They were randomly divided into 2 groups, with 5 mice in each group. All animal procedures were performed in strict accordance with the principles of the Institutional Animal Care and Use Committee of the Affiliated Cancer Hospital of Zhengzhou University. Eca-109 cells (6 × 10^6^) infected with lentivirus vectors carrying sh-THAP9-AS1 or negative control (sh-NC) were suspended in 200 μl normal saline and subcutaneously inoculated into the left flank of nude mice (*n* = 5/group). When the tumors were visible, tumor sizes were routinely measured with a vernier caliper every 5 days. The tumor volumes were calculated using the formula: V (mm^3^) = Length × Width^2^ × 0.5. At the end of the experiment, xenograft tumors were excised for further assay. Researchers who were blinded to the control and treatment groups carried out the tumor measurements and statistical analysis.

### Immunohistochemistry (IHC)

Tumor tissues were fixed with 4% paraformaldehyde, embedded in paraffin, and sectioned into 3–4 μm thick slices. After incubation with primary antibody against Ki-67 (Cell Signaling Technology, Beverly, MA, USA; Cat. #12202) or PCNA (Cell Signaling Technology; Cat. #2586) at 4 °C overnight, the tissue sections were further treated with HRP-conjugated secondary antibody (Cell Signaling Technology; Cat. #8114 S) for 1 h at room temperature. Subsequently, the tissue sections were stained with diaminobenzidine and hematoxylin, followed by a light microscope to capture the images.

### Statistical analysis

All data assays were done using GraphPad Prism 7 (GraphPad Software, Inc., La Jolla, CA, USA). The measurement data were expressed as mean ± standard deviation (SD) representing the results of 3 independent experiments. Two-tailed student’s *t*-test or one-way analysis of variance (ANOVA) followed by Bonferroni post-test was utilized to compare the difference between clinical cohort groups or cell groups. Normal distribution and similar variances were assumed. Pearson χ2 test was used to explore the association of THAP9-AS1 expression with clinicopathological features of ESCC patients. The median value of THAP9-AS1 expression was set as the cut-off value to stratify patients into high and low expression groups. The probability of survival was determined with the Kaplan-Meier method and compared using the log-rank test. When the *P-*value was less than 0.05, results were considered statistically significant.

## Results

### THAP9-AS1 is highly expressed in ESCC and predicts a poor prognosis

**Fig. 1:**
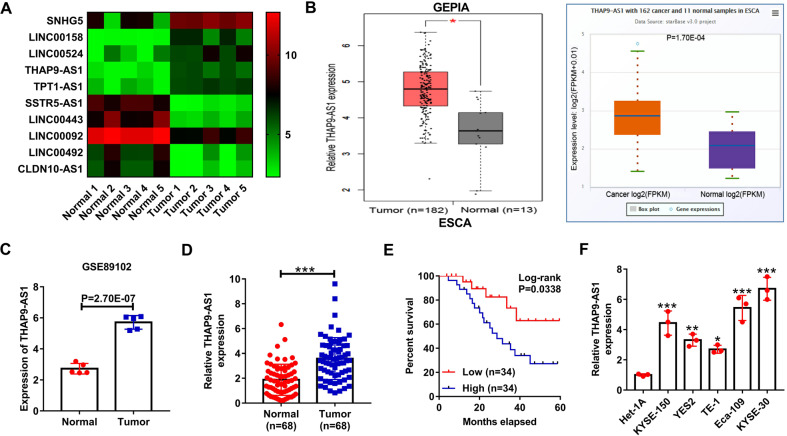
THAP9-AS1 is upregulated and associated with a poor prognosis in ESCC. **A** 5 upregulated and 5 downregulated lncRNAs were displayed by heat map according to microarray dataset (GSE89102) containing 5 ESCC tumor tissues neighboring non-cancerous tissues. **B** The expression profile of THAP9-AS1 in esophageal carcinoma from TCGA by using GEPIA and starBase. **C** Microarray data from GSE53625 showed the expression difference of THAP9-AS1 in ESCC tumor tissues and adjacent normal tissues. **D** qRT-PCR analysis was performed to measure the expression of THAP9-AS1 in tumor tissues and corresponding nonneoplastic tissues from 68 ESCC patients. **E** Kaplan-Meier survival curve was utilized to evaluate the correlation between THAP9-AS1 expression and clinical outcome of ESCC patients. **F** Expression of THAP9-AS1 in ESCC cell lines (KYSE-150, YES2, TE-1, Eca-109, KYSE-30) and normal esophageal epithelial cell line Het-1A was determined by qRT-PCR. *P < 0.05, **P < 0.01, ***P < 0.001.

With the purpose of identifying abnormally expressed lncRNAs in ESCC, public microarray profiling datasets (GSE89102) containing 5 ESCC tumor tissues and adjacent non-neoplastic tissues were downloaded from the NCBI/GEO database. Five upregulated and five downregulated lncRNAs were displayed in the heatmap based on the fold change and unknown characteristics (Fig. 1A). In these upregulated lncRNAs, only THAP9-AS1 expression was significantly increased in esophageal carcinoma tumor tissues when compared to normal tissues in both GEPIA (http://gepia.cancer-pku.cn/) and starBase (http://starbase.sysu.edu.cn/) websites (Fig. 1B and Supplementary Fig. S1A–D). Hence, THAP9-AS1 was selected as a study subject. Based on the data from GSE89102, we observed a significant rise of THAP9-AS1 expression in ESCC tumor tissues compared with neighboring normal tissues (Fig. 1C). Consistently, THAP9-AS1 expression was confirmed to be higher in tumor tissue samples than that in corresponding non-cancerous specimens from 68 ESCC patients (Fig. 1D). Moreover, THAP9-AS1 expression was positively correlated with tumor size (*P* = 0.015), TNM stage (*P* = 0.013), and lymph node metastasis (*P* = 0.028) (Supplementary Table S1). Kaplan-Meier survival curves displayed a lower survival rate in ESCC patients expressing a high level of THAP9-AS1 (Fig. 1E). In addition, there was a significant elevation of THAP9-AS1 expression in ESCC cell lines (KYSE-150, YES2, TE-1, Eca-109, KYSE-30) as compared to that in normal human esophageal epithelial cell line Het-1A, especially in Eca-109 and KYSE-30 (Fig. 1F). These data suggested that THAP9-AS1 expression was abnormally upregulated in ESCC.

### Knockdown of THAP9-AS1 inhibits cell growth, migration, and invasion, while induces apoptosis in ESCC

**Fig. 2:**
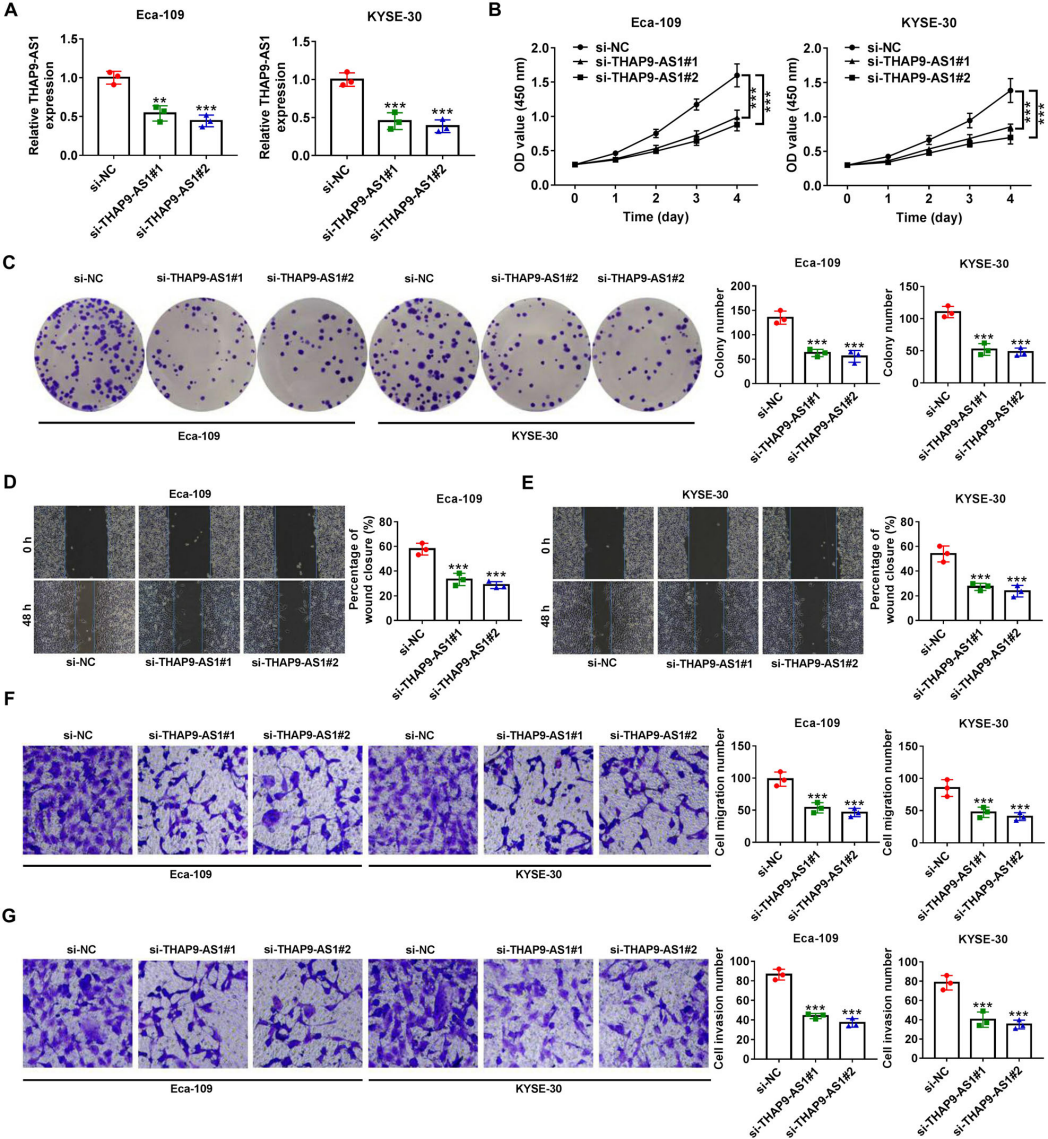
THAP9-AS1 promotes ESCC cell proliferation, migration, and invasion in vitro. **A** qRT-PCR was applied to detect the transfection efficiency of si-RNAs targeting THAP9-AS1 (si-THAP9-AS1#1 or si-THAP9-AS1#2) in Eca-109 and KYSE-30 cells. **B** and **C** CCK-8 and colony formation assays were used to assess the effect of THAP9-AS1 depletion on cell proliferation. **D** and **E** A wound-healing assay were conducted to detect the influence of THAP9-AS1 knockdown on ESCC cell migration capability. **F** and **G** Transwell assays were employed to evaluate the migration and invasion abilities of Eca-109 and KYSE-30 cells with THAP9-AS1 knockdown. **P < 0.01, ***P < 0.001.

To clarify the biological significance of THAP9-AS1 in ESCC progression, siRNAs specifically targeting THAP9-AS1 including si-THAP9-AS1#1 and si-THAP9-AS1#2 were transfected into Eca-109 and KYSE-30 cells. The transfection efficiency was determined by qRT-PCR, with the results showing a significant decrease of THAP9-AS1 expression in Eca-109 and KYSE-30 cells (Fig. 2A). As demonstrated by CCK-8 and clonogenic assays, silencing of THAP9-AS1 resulted in significant suppression of cell proliferation and colony-forming capability (Fig. 2B, C). Flow cytometry assays displayed a significant increase of apoptotic rate in Eca-109 and KYSE-30 cells after transfection with si-THAP9-AS1#1 or si-THAP9-AS1#2 (Supplementary Fig. S2A). Subsequently, we further made an investigation on the regulatory effect of THAP9-AS1 on ESCC cell metastasis. Wound healing assays manifested that depletion of THAP9-AS1 lowered cell motility (Fig. 2D, E). Accordingly, transwell assays uncovered a significant decline of cell migration and invasion in Eca-109 and KYSE-30 cells with low expression of THAP9-AS1 (Fig. 2F, G). The above results indicated the promotive role of THAP9-AS1 in ESCC cell proliferation, migration, and invasion.

### THAP9-AS1 acts as a molecular sponge for miR-133b in ESCC cells

**Fig. 3:**
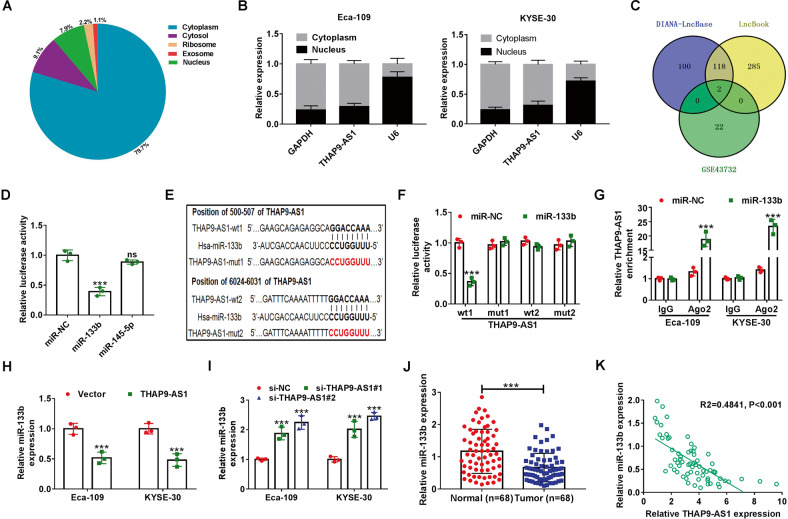
THAP9-AS1 serves as a sponge for miR-133b in ESCC cells. **A** The cellular location of THAP9-AS1 was predicted by the online tool lncLocator. **B** The relative expression of THAP9-AS1 in the cytoplasm and nucleus of ESCC cells was determined via subcellular fractionation and qRT-PCR assays. **C** By retrieving the GEO dataset (GSE43732) and using algorithms (DIANA-LncBase and LncBook), two downregulated miRNAs (miR-133b and miR-145-5p) in ESCC were identified to be able to potentially interact with THAP9-AS1. **D** Dual-luciferase reporter assay was used to evaluate the effect of miR-133b or miR-145-5p on the luciferase activity of THAP9-AS1-wt reporter in Eca-109 cells. **E** The binding sequences of THAP9-AS1 on miR-133b and corresponding mutant sequences. **F** Dual-luciferase reporter experiments were performed in Eca-109 and KYSE-30 cells co-transfected with miR-NC or miR-133b and THAP9-AS1-wt1 or THAP9-AS1-mut1/THAP9-AS1-wt2 or THAP9-AS1-mut2. **G** miR-NC-transfected or miR-133b-transfected ESCC cells were subjected to anti-Ago2 RIP assay, then qRT-PCR was used to measure the abundance of THAP9-AS1 enrichment in immunoprecipitates. **H**, **I** qRT-PCR assays were carried out to measure the expression of miR-133b in Eca-109 and KYSE-30 cells upon THAP9-AS1 overexpression or knockdown. **J** miR-133b expression in tumor tissues and adjacent normal tissues from 68 ESCC patients was measured by qRT-PCR analysis. **K** Expression correlation analysis between miR-133b and THAP9-AS1 in ESCC tumor tissues. ***P < 0.001.

In order to unearth the action mechanism by which THAPH-AS1 facilitates ESCC progression, we firstly made a prediction of the subcellular location of THAP9-AS1 with an online tool lncLocator (http://www.csbio.sjtu.edu.cn/bioinf/lncLocator/). As depicted in Fig. 3A, THAP9-AS1 mainly existed in the cytoplasm (79.7%) and cytosol (7.9%). Subcellular fractionation experiments were performed in ESCC cells to verify this forecast result. In Eca-109 and KYSE-30 cells, it was turned out that THAP9-AS1 expression in the cytoplasm was much more than that in the nucleus (Fig. 3B). To our knowledge, cytoplasmic lncRNAs are able to serve as competing endogenous RNAs (ceRNAs) to sequester miRNAs and thereby protect their target mRNAs from being attacked^19^. In view of the cytoplasmic localization of THAP9-AS1, we wondered whether the carcinogenicity of THAP9-AS1 in ESCC was ascribed to the ceRNA mechanism. By using DIANA-LncBase v2.0 and LncBook algorithm, a total of 120 candidate miRNAs possessing the potential binding sites of THAP9-AS1 were predicted. By comparing the predicted miRNAs with the downregulated miRNAs in ESCC from the GSE43732 dataset, only 2 miRNAs (miR-133b and miR-145-5p) were identified in the intersection of the Venn diagram (Fig. 3C). As presented in Supplementary Fig. S3A, low expression of miR-133b and miR-145-5p was observed in ESCC tumor tissues compared to adjacent normal tissues. Consistently, the starBase website also showed significant downregulation of miR-133b and miR-145-5p in esophageal carcinoma tumor tissues compared with normal samples (Supplementary Fig. S3B). Moreover, the luciferase activity of THAP9-AS1-wt reporter in Eca-109 cells was suppressed by miR-133b rather than miR-145-5p (Fig. 3D). Thus, miR-133b was chosen for further assay. There are two binding sites on THAP9-AS1 (500–507 and 6024–6031) for miR-133b (Fig. 3E). To validate the actual binding between THAP9-AS1 and miR-133b, dual-luciferase reporter assays were conducted in Eca-109 cells after transfection with miR-NC or miR-133b and THAP-AS1-wt1 or THAP-AS1-mut1/THAP-AS1-wt2 or THAP-AS1-mut2. As a result, overexpression of miR-133b inhibited the luciferase activity of THAP-AS1-wt1 reporter compared with the miR-NC group, however, no change was found in the luciferase activity of reporter with THAP-AS1-mut1, THAP-AS1-wt2, or THAP-AS1-mut2 between miR-NC and miR-133b group (Fig. 3F), suggesting the binding of miR-133b on THAP9-AS1 is at site 1 (position of 500–507). Subsequent RIP assay found that compared with the miR-NC group, much more THAP9-AS1 was precipitated with Ago2 antibody in miR-133b group (Fig. 3G). Moreover, miR-133b expression was inhibited by the overexpression of THAP9-AS1, while was promoted due to the depletion of THAP9-AS1 (Fig. 3H, I). Furthermore, miR-133b expression was lower in 68 ESCC tumor tissues than that in adjacent non-cancerous tissues (Fig. 3J). And, miR-133b was inversely correlated with THAP9-AS1 expression in ESCC tumor tissues (Fig. 3K). All these data indicated that THAP9-AS1 could repress miR-133b expression, at least in part, through direct binding in ESCC cells.

### THAP9-AS1 drives malignant cell phenotypes in ESCC by downregulating miR-133b

**Fig. 4:**
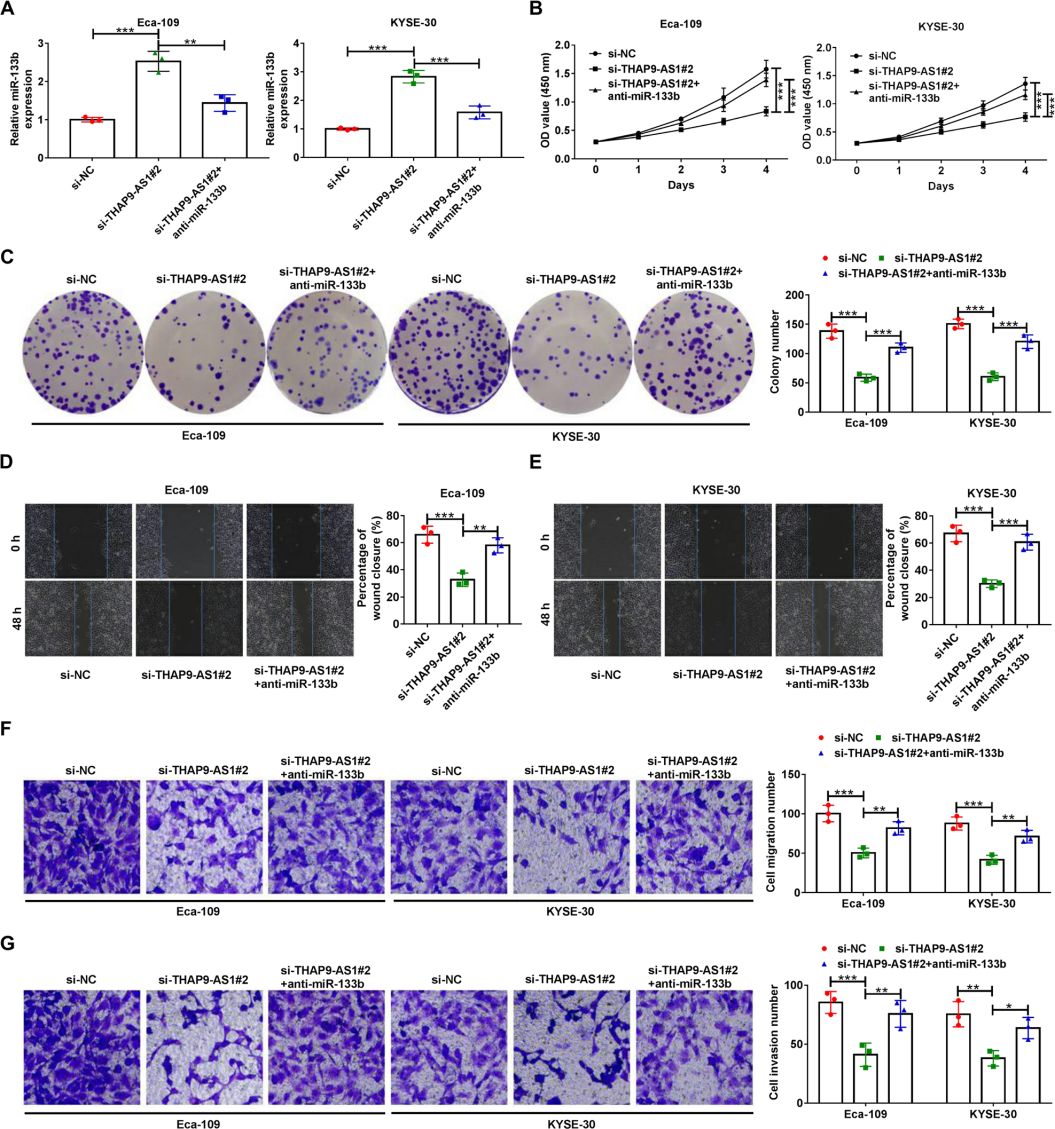
THAP9-AS1 exerts tumor-promoting effects in ESCC through sponging miR-133b. **A**–**G** Eca-109 and KYSE-30 cells were transfected with si-NC, si-THAP9-AS1#2 or si-THAP9-AS1#2+anti-miR-133b, followed by qRT-PCR assay of miR-133b expression (**A**), CCK-8 assay of cell viability (**B**), colony formation assay (**C**), wound healing assay of cell migration (**D** and **E**), and transwell assay of cell migration and invasion (**F** and **G**). *P < 0.05, **P < 0.01, ***P < 0.001.

To figure out whether THAP9-AS1 exerts oncogenic property in ESCC depending on miR-133b, si-THAP9-AS1#2-transfected Eca-109 and KYSE-30 cells were introduced with miR-133b inhibitor. As exhibited in Fig. 4A, a si-THAP9-AS1-mediated increase of miR-133b expression was evidently reversed by transfection with anti-miR-133b. Moreover, downregulation of miR-133b partially abrogated the inhibitory effects of THAP9-AS1 knockdown on cell growth and colony-forming ability (Fig. 4B, C). Also, si-THAP9-AS1-induced apoptosis was abated in the presence of miR-133b inhibitor (Supplementary Fig. S2B). As for metastatic potential, the suppression of cell motility caused by THAP9-AS1 silencing was effectively eliminated due to the decrease of miR-133b expression (Fig. 4D, E). Likewise, cell migration and invasion capability depressed by THAP9-AS1 depletion were significantly restored following the suppression of miR-133b expression (Fig. 4F, G). Collectively, miR-133b was a downstream mediator of THAP9-AS1-induced proliferative and metastatic activity.

### THAP9-AS1 increases SOX4 expression through sponging miR-133b

**Fig. 5:**
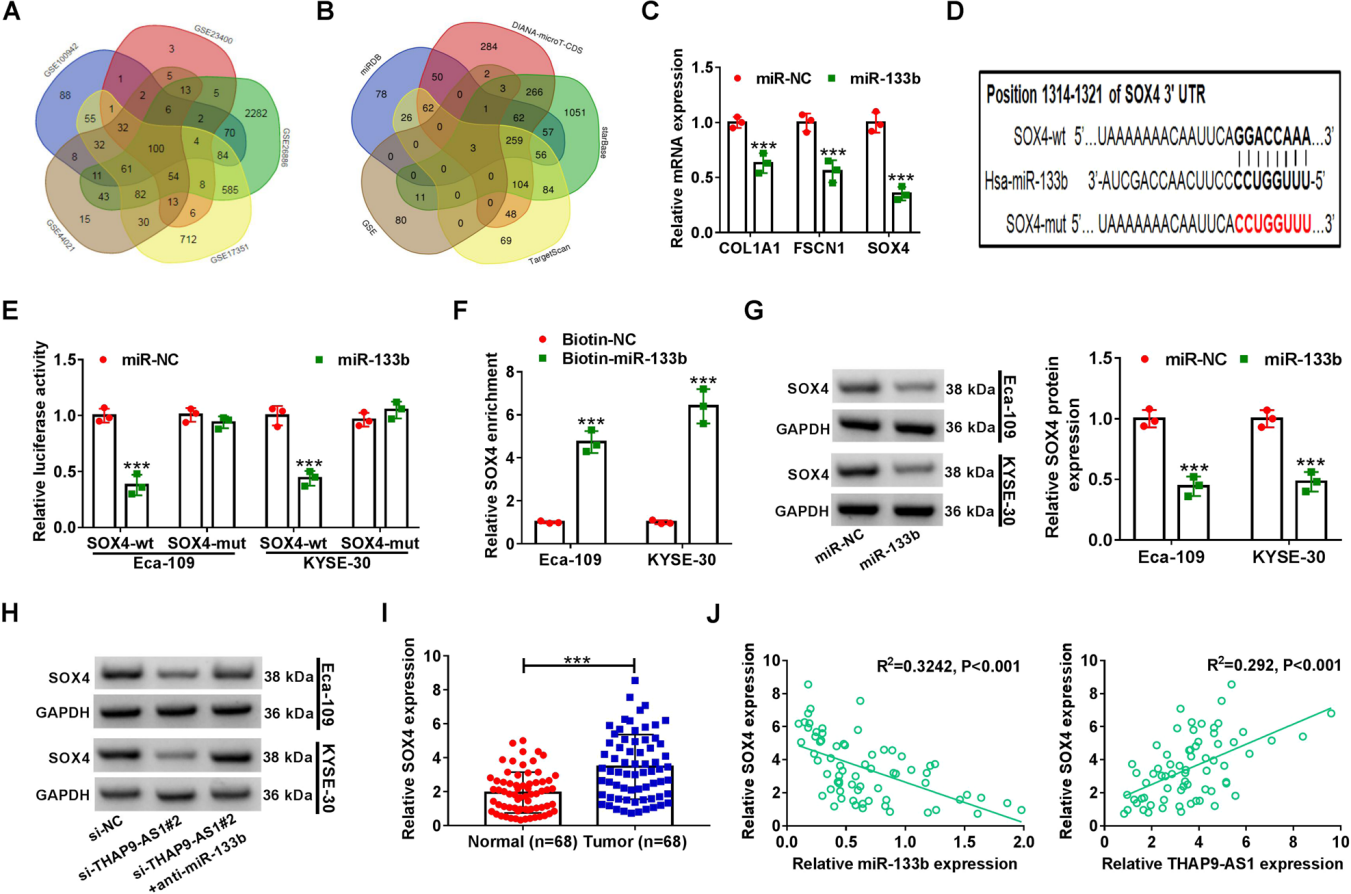
THAP9-AS1 increases SOX4 expression through competitively binding to miR-133b in ESCC cells. **A** A total of 100 upregulated genes were identified in ESCC from 5 different ESCC patient cohorts (GSE100942, GSE23400, GSE26886, GSE17351, GSE44021) based on the criteria of log2 fold change ≥1. **B** Using 4 freely available databases (TargetScan, miRDB, DIANA-microT-CDS, starBase), three genes (COL1A1, FSCN1, and SOX4) that contain the potential binding sites of miR-133b were filtered out among these 100 upregulated candidates. **C** qRT-PCR was used to uncover the effect of miR-133b overexpression on the mRNA level of SOX4, FSCN1, and COL1A1 in Eca-109 cells. **D** The wild-type complementary sequences of SOX4-3′UTR on miR-133b and corresponding mutant sequences were shown. **E** Dual-luciferase reporter assays were performed in both Eca-109 and KYSE-30 cells after cotransfection with miR-NC or miR-133b and SOX4-wt or SOX4-mut reporter. **F** Biotinylated RNA pull-down assays were used to demonstrate the binding between miR-133b and SOX4. **G** Western blot assays were used to measure the protein expression of SOX4 in miR-NC-overexpressing or miR-133b-overexpressing ESCC cells. **H** The protein level of SOX4 was determined by western blot in Eca-109 and KYSE-30 cells transfected with si-NC, si-THAP9-AS1#2, or si-THAP9-AS1#2+anti-miR-133b. **I** Expression difference of SOX4 mRNA in tumor tissues and corresponding para-carcinoma tissues from a cohort of 68 ESCC patients. **J** Correlation analysis between SOX4 mRNA expression and miR-133b or THAP9-AS1 in 68 ESCC tumor specimens. ***P < 0.001.

Next, we further delved into the underlying mechanism of miR-133b involved in ESCC. According to microarray mRNA expression profile datasets (GSE100942, GSE23400, GSE26886, GSE17351, GSE44021), a total of 100 upregulated genes were identified in ESCC by Venn diagram (Fig. 5A). By searching in 4 open-source software (TargetScan, miRDB, DIANA-microT-CDS, starBase), there only 3 genes (COL1A1, FSCN1, and SOX4) were predicted to harbor the binding sites on miR-133b among these 100 candidates (Fig. 5B). GEPIA and starBase web tools also displayed higher expression of SOX4, FSCN1, and COL1A1 in esophageal carcinoma tumor tissues than that in normal tissues (Supplementary Fig. S4A, B). Subsequently, we measured the effect of miR-133b on these gene expressions in Eca-109 cells. As demonstrated by qRT-PCR, SOX4 presented the most significant decline due to miR-133b overexpression (Fig. 5C). Hence, SOX4 was listed as the focus for further investigation. To confirm the binding between miR-133b and SOX4, luciferase reporter vectors possessing either wild type or mutant SOX4-3′UTR, in which there existed the binding sequences of miR-133b or corresponding mutant sequences, were constructed (Fig. 5D). As shown in Fig. 5E, compared with miR-NC, miR-133b overexpression significantly repressed the luciferase activity SOX4-wt reporter but not SOX4-mut reporter in Eca-109 and KYSE-30 cells. RNA pull-down assays found that Biotin-miR-133b captured much more SOX4 mRNA than Biotin-NC (Fig. 5F). Moreover, western blot assays showed that miR-133b mimics resulted in a significant decrease of SOX4 protein level in both Eca-109 and KYSE-30 cells (Fig. 5G). The aforementioned results indicated SOX4 as a direct target of miR-133b. And more notably, knockdown of THAP9-AS1 also repressed the protein expression of SOX4, while this effect was obviously countervailed by miR-133b inhibitor (Fig. 5H). In addition, a significantly higher SOX4 mRNA expression was observed in tumor tissues than that in adjacent normal tissues from our cohort of 68 ESCC patients (Fig. 5I). Furthermore, SOX4 expression was negatively associated with miR-133b expression, while positively correlated with THAP9-AS1 level in ESCC tumor tissues (Fig. 5J). All these data supported the conclusion that THAP9-AS1 could competitively bind to miR-133b, leading to the upregulation of SOX4 expression.

### Suppression of cell biological malignant behavior caused by THAP9-AS1 depletion is abated following the upregulation of SOX4

**Fig. 6:**
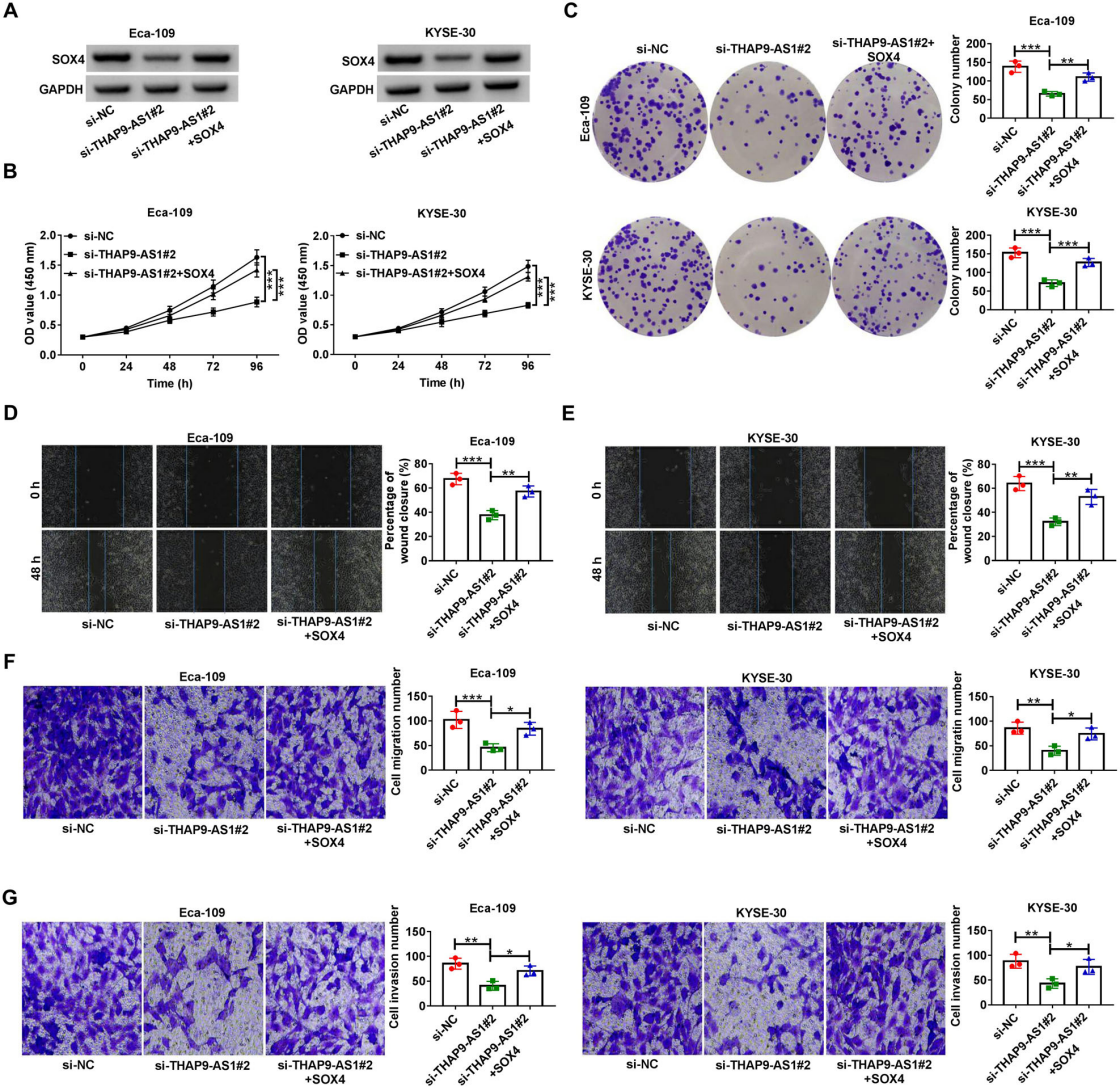
THAP9-AS1 facilitates ESCC progression in vitro via modulating SOX4 expression. **A**–**G** Eca-109 and KYSE-30 cells were transfected with si-NC, si-THAP9-AS1#2 or si-THAP9-AS1#2 + SOX4, followed by western blot assay of SOX4 protein expression (**A**), CCK-8 assay of cell viability (**B**), colony formation assay (**C**), wound healing assay of cell migration (**D** and **E**), and transwell assay of cell migration and invasion (**F** and **G**). *P < 0.05, **P < 0.01, ***P < 0.001.

To gain insight into whether the carcinogenesis of THAP9-AS1 in ESCC was associated with SOX4, the SOX4-overexpression vector was transfected into Eca-109 and KYSE-30 cells with THAP9-AS1 knockdown. Western blot assay showed that si-THAP9-AS1-induced decrease of SOX4 protein expression was reversed by transfection with pcDNA-SOX4 (Fig. 6A). Moreover, the lowered proliferation ability conferred by THAP9-AS1 knockdown was effectively restored by SOX4 overexpression (Fig. 6B, C). Also, si-THAP9-AS1-induced apoptosis was attenuated in the presence of pcDNA-SOX4 (Supplementary Fig. S2C). Meanwhile, ectopic expression of SOX4 partially counteracted the inhibitory effects of THAP9-AS1 silencing on cell motility (Fig. 6D and E). Similarly, the suppression of cell migration and invasion induced by THAP9-AS1 depletion was greatly disrupted due to the increase of SOX4 expression (Fig. 6F, G). Taken together, THAPP9-AS1 facilitated ESCC progression in vitro via modulating SOX4 expression.

### Transcription factor SOX4 binds to the promoter of THAP9-AS1 to activate its transcription

**Fig. 7:**
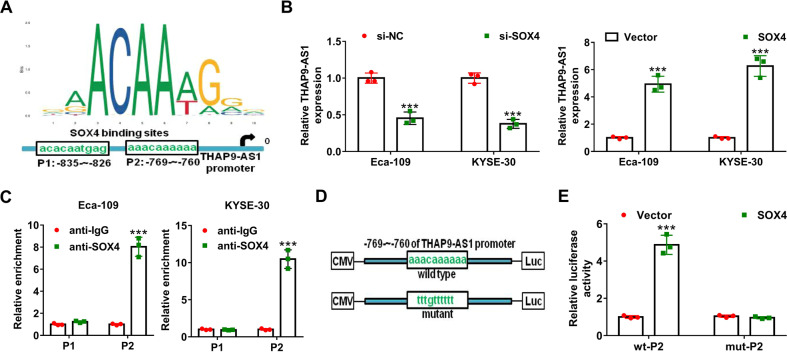
SOX4 transcriptionally activates THAP9-AS1 in ESCC cells. **A** JASPAR (http://jaspar.genereg.net/) was used to predict the binding sites of transcription factor SOX4 on THAP9-AS1 promoter region. **B** qRT-PCR assays were used to detect the expression of THAP9-AS1 in Eca-109 and KYSE-30 cells with SOX4 knockdown or overexpression. **C** ChIP assay was applied to detect the binding affinity of SOX4 with THAP9-AS1 promoter at the two predicted sites (P1 and P2) by using antibodies against SOX4 or IgG. **D** Regarding the P2 binding sites (−769 ∼ −760) of SOX4 on the THAP9-AS1 promoter, wild-type, and mutant luciferase vectors was constructed accordingly. **E** Dual-luciferase reporter assays were performed in HEK-293T after transfection with wt-P2 or mut-P2 and vector or pcDNA-SOX4. ***P < 0.001.

**Fig. 8:**
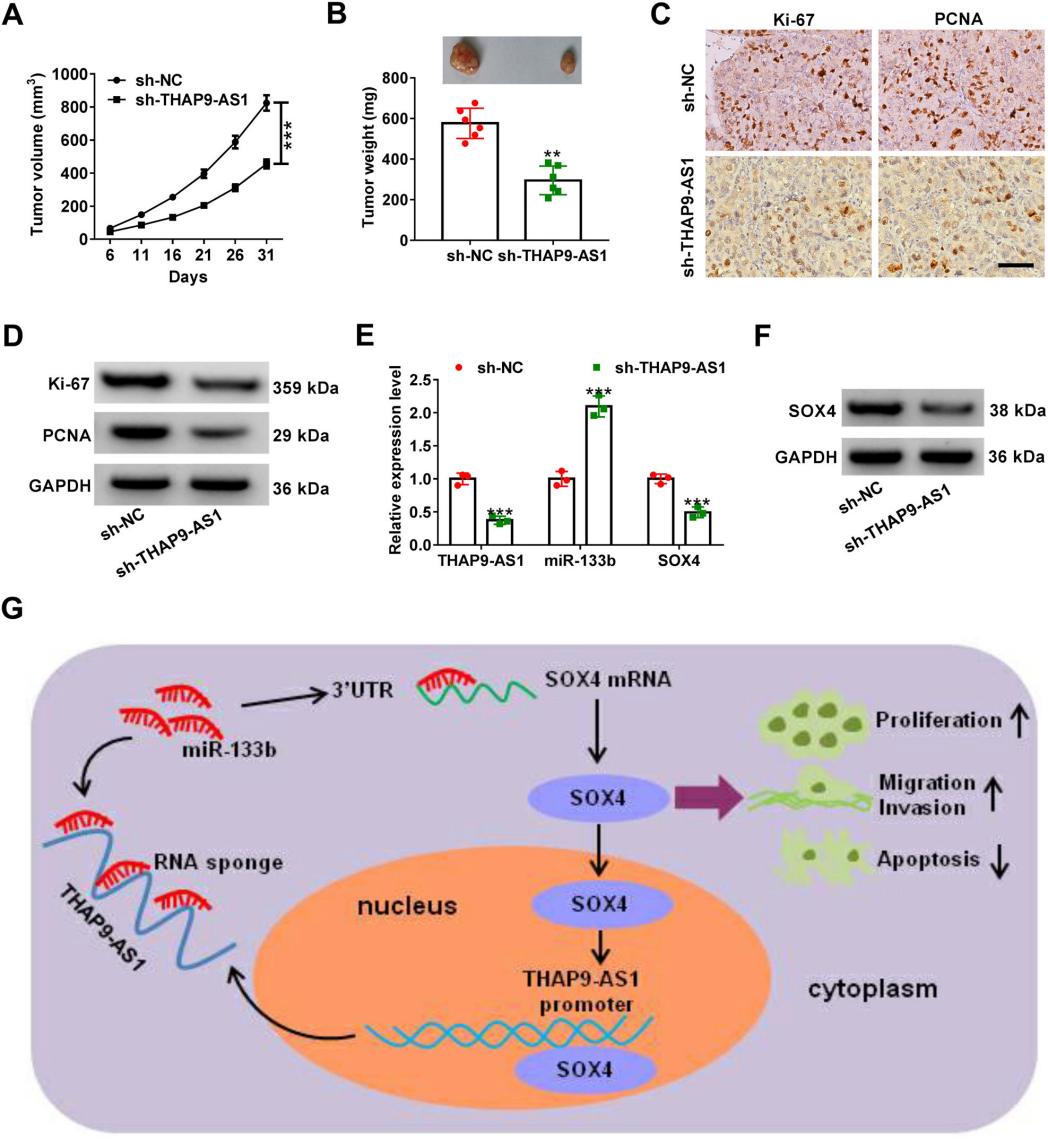
THAP9-AS1 accelerates ESCC tumor growth in vivo. Eca-109 cells infected with lentivirus vectors carrying sh-THAP9-AS1 or negative control (sh-NC) were inoculated into nude mice. **A** Growth curve of xenografts from mice in sh-NC and sh-THAP9-AS1 group. **B** Quantification of tumor weight in mice at 31 days after injection. **C** and **D** IHC staining and western blot assay were used to determine the protein expression of Ki-67 and PCNA in excised tumors. **E** qRT-PCR assay was utilized to evaluate the expression of THAP9-AS1, miR-133b, and SOX4 mRNA in resected tumors. **F** Western blot assay of SOX4 protein level in xenografts from mice. **G** A schematic illustration shows that SOX4-induced THAP9-AS1 promotes cell proliferation, migration, and invasion by sponging miR-133b and upregulating SOX4, depicting a THAP9-AS1/miR-133b/SOX4 positive feedback loop that contributes to ESCC progression. ***P < 0.001.

As reported, transcription factors could participate in the modulation of abnormal expression of lncRNAs in human malignancies^20,21^. With the application of the JASPAR (http://jaspar.genereg.net) website, SOX4 was found to harbor two major binding sites on the promoter region of THAP9-AS1 (Fig. 7A). Moreover, THAP-AS1 expression was suppressed by SOX4 knockdown, while was enhanced due to SOX4 overexpression (Fig. 7B). To confirm the binding between SOX4 and THAP9-AS1 promoter, a ChIP assay was performed. The results manifested the SOX4 occupancy at the P2 site (−769 ∼ −760) rather than the P1 site (−835 ∼ −826) on the THAP9-AS1 promoter (Fig. 7C). Next, the wild type or mutant P2 sequence was, respectively, constructed into the luciferase reporter plasmids to perform dual-luciferase reporter experiments (Fig. 7D). As shown in Fig. 8E, enforced expression of SOX4 led to a significant increase of wt-P2 promoter activity but not mut-P2 promoter (Fig. 7E). Overall, SOX4 induced the transcription activation of THAP9-AS1.

### THAP9-AS1 promotes ESCC tumorigenesis in vivo

To investigate the effect of THAP9-AS1 on ESCC tumorigenesis in vivo, subcutaneous injection of Eca-109 cells carrying sh-THAP9-AS1 or sh-NC was implemented in nude mice. The results revealed that knockdown of THAP9-AS1 significantly lowered tumor growth and tumor weight (Fig. 8A, B). IHC analysis found a decrease of Ki-67 and PCNA expression in xenograft tumor tissues with THAP9-AS1 knockdown compared to the sh-NC group (Fig. 8C). Consistently, the western blot assay also showed that Ki-67 and PCNA protein expression was reduced in tumor tissues derived from sh-THAP9-AS1-transfected cells (Fig. 8D). As demonstrated by qRT-PCR, depletion of THAP9-AS1 resulted in a decline of THAP-AS1 and SOX4 mRNA expression, while an increase of miR-133b in excised tumor tissues (Fig. 8E). In addition, SOX4 protein expression was lower in THAP9-AS1-depletion tumor tissues than that in the sh-NC group (Fig. 8F). To be concluded, THAP9-AS1 contributed to ESCC tumor growth in vivo.

## Discussion

Although the incidence of ESCC shows a declining trend in most countries, it is still high in many populations^22^. A growing body of researches illuminates that aberrant expression of lncRNAs plays a crucial role in different physiological and pathological processes of human cancers^23^. However, there are lots of lncRNAs to be functionally characterized and mechanistically elucidated in order to search for potential targets for cancer intervention. Through mining the data from GSE89102 containing 5 pairs of ESCC tumor tissues and adjacent normal tissues, we focused on the biological significance of THAP9-AS1, as well as its underlying mechanism in ESCC. This study indicates that THAP9-AS1 contributes to ESCC progression by sponging miR-133b to upregulate SOX4, which in turn transcriptionally activates THAP9-AS1. Together, our findings highlight a positive feedback loop of THAP9-AS1/miR-133b/SOX4 in ESCC, providing a certain theoretical basis for employing THAP9-AS1 as a potential prognostic biomarker and therapeutic target for ESCC patients.

In recent years, increasing lncRNAs are identified to be associated with ESCC occurrence and progression^24^. In this study, we found that THAP9-AS1 expression was increased in ESCC tumor tissues and cells. Moreover, high THAP9-AS1 expression was positively correlated to tumor size, TNM stage, lymph node metastasis, and poor prognosis of ESCC patients. Functionally, silencing of THAP9-AS1 resulted in a suppression of cell proliferation, migration, and invasion, while induced apoptosis. Also, depletion of THAP9-AS1 inhibited xenograft growth in vivo. These data suggested the carcinogenic effect of THAP9-AS1 in ESCC. Interestingly, there also are documents reporting the carcinogenic effects of THAP9-AS1 in other types of solid tumors. Li et al. revealed the extensive overexpression of THAP9-AS1 in PDAC, and high THAP9-AS1 expression predicted a poor clinical outcome^15^. Moreover, knockdown of THAP9-AS1 decreased cell proliferation and colony-forming capacity in vitro and inhibited tumor growth in vivo^15^. Also, THAP9-AS1, induced by *H. pylori*, was highly expressed in gastric cancer tissues and facilitated cell proliferation and migration in vitro^16^. Our study, for the first time, demonstrated the dysregulation of THAP9-AS1 in ESCC and its stimulative effects on cell growth and metastasis. All these data indicated the potential of THAP9-AS1 as a prognostic biomarker and therapeutic target in lncRNA-based cancer therapy.

Mounting evidence reveals that cytoplasmic lncRNAs can act as miRNA sponges to prevent miRNA-mediated translational repression of target mRNA^25,26^. In line with the prediction result from lncLocator software, THAP9-AS1 was experimentally validated to be predominantly located in the cytoplasm by subcellular fractionation assays. Hence, THAP9-AS1 was conjectured to exert regulatory effects in ESCC through a similar action mechanism. By using a series of bioinformatics tools, luciferase reporter, and RIP experiments, miR-133b was certified as a bona fide target of THAP9-AS1. A previous review summarized that miR-133b could function as a tumor suppressor or promoter in various malignant tumors by affecting cell proliferation, apoptosis, migration, motility, energy metabolism, and radiochemotherapy resistance^27^. To date, several documents demonstrated miR-133b exerted an antineoplastic property in ESCC. For example, Zeng et al. reported that overexpression of miR-133b repressed cell proliferation, migration, and invasion in ESCC cells via inactivating MAPK/ERK and PI3K/AKT signaling pathways by targeting EGFR^28^. Huang et al. unveiled that miR-133b suppressed cell growth and induced apoptosis during ESCC progression through regulating cullin 4B expression^29^. Zhu et al. disclosed that miR-133b slowed down the tumor growth and lung metastases in ESCC via regulating EGFR/ITGB4/FAK/Grb2 signaling pathway^30^. On this basis, we herein further explored whether miR-133b was responsible for the inhibition of THAP9-AS1 knockdown on ESCC cell malignant phenotypes by rescue experiments. As a result, the suppressive effects of THAP9-AS1 silencing on cell proliferation, migration, and invasion were partly abolished by the miR-133b inhibitor. It is thus possible that THAP9-AS1 promoted ESCC development by sponging miR-133b.

SOX4, a member of the SOX (Sry-related high-mobility group (HMG) box) family of transcription factors, is reported to drive cancer development and progression through endowing cancer cells with survival, migratory, and invasive abilities^31^. A large body of evidence demonstrates that SOX4 is generally amplified and upregulated in nearly all major human cancers, and acts as an oncogene by promoting stemness, cancer cell proliferation, angiogenesis, migration, EMT, and metastasis via affecting downstream genes in cancer-associated signaling pathways^32^. For instance, SOX4 overexpression facilitated tumor aggressiveness in bladder cancer by regulating cellular invasion via repressing WNT5a expression^33^. SOX4 was highly expressed in renal cell carcinoma tissues and cell lines, and SOX4 accelerated cell migration and invasion by inducing EMT via activating AKT signaling cascade^34^. SOX4 contributed to cell growth and metastasis in breast cancer in vitro and in vivo by binding to the CXCR7 promoter to enhance its transcription^35^. SOX4 was overexpressed in gastric cancer, and enforced expression of SOX4 promoted TGF-β-induced EMT and stem cell characteristics in gastric cancer through activation of the Wnt pathway^36^. Evidence to support this notion is also present in ESCC. Han et al. found that SOX4 was upregulated, and knockdown of SOX4 inhibited cell proliferation and enhanced doxorubicin-induced cell senescence^37^. In this study, SOX4 was identified as a downstream target of miR-133b in ESCC cells. Mechanistically, THAP9-AS1 could serve as a sponge for miR-133b to relieve its inhibition on SOX4 expression. More importantly, the antitumor property induced by THAP9-AS1 loss was reversed by the restoration of SOX4 expression. As noted above, it was concluded that THAP9-AS1 promoted tumorigenicity and metastasis in ESCC by positively regulating SOX4 expression. Another finding in our study was that SOX4 could bind to the promoter region of THAP9-AS1 to activate its transcription. As demonstrated by Li et al, THAP9-AS1 facilitated PDAC growth through sponging miR-484 and interacting with YAP^15^. Our study elucidated a novel regulatory mechanism of THAP9-AS1, a positive feedback loop of THAP9-AS1/miR-133b/SOX4, contributing to ESCC development.

All in all, THAP9-AS1 is upregulated in ESCC tissues and cells. Functionally, depletion of THAP9-AS1 suppresses cell proliferation, migration, and invasion in vitro and lowers tumor growth in vivo. Mechanistically, THAP9-AS1 sponges miR-133b to increase the expression of SOX4, which in turn, binds to the THAP9-AS1 promoter to activate its transcription. Our findings elucidate a positive feedback loop of THAP9-AS1/miR-133b/SOX4 in facilitating ESCC progression (Fig. 8G), providing a better understanding of lncRNA-based target treatment and highlighting a valuable therapeutic strategy for ESCC. Nonetheless, further clinical researches are warranted for the immediate human application of THAP9-AS1 to combat ESCC.

## Supplementary information

Supplementary Figures

Supplementary Tables

Detailed Attribution of Authorship

Reproducibility Checklist Forms
