# Supplementary material for: THAP9-AS1/miR-133b/SOX4 positive feedback loop facilitates the progression of esophageal squamous cell carcinoma
Source: Cell Death Dis. 2021 Apr 14;12(4):401. doi: 10.1038/s41419-021-03690-z (PMC8046801; doi:10.1038/s41419-021-03690-z)
Supplement: Supplementary file 1 — Supplementary Figures [file 41419_2021_3690_MOESM1_ESM.docx]

**Supplementary Figures**

**
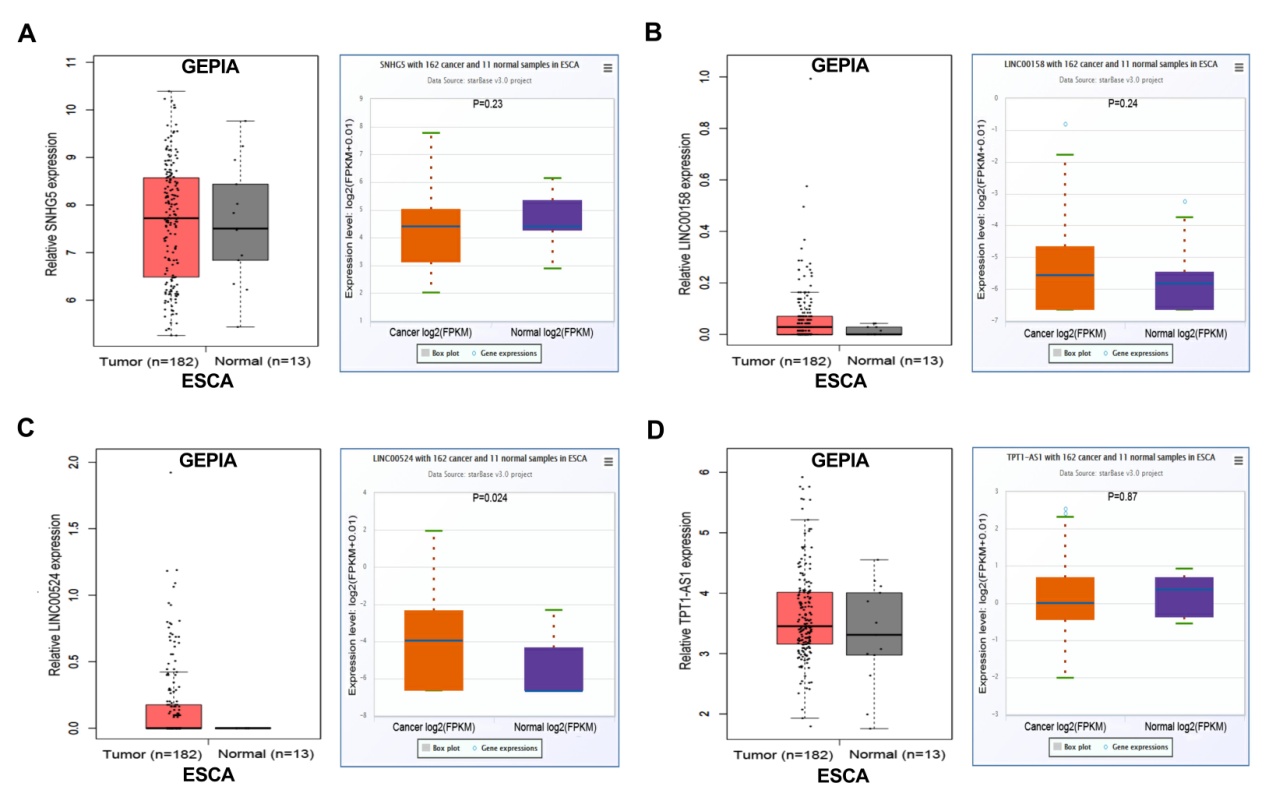
**

**Supplementary Figure S1. Expression of 4 up-regulated lncRNAs identified from GSE89102 in** [**esophageal carcinoma**](javascript:;) **was analyzed by GEPIA (**[**http://gepia.cancer-pku.cn/**](http://gepia.cancer-pku.cn/)**) and starBase (**[**http://starbase.sysu.edu.cn/**](http://starbase.sysu.edu.cn/)**) online tools.** (A) SNHG5 expression in [esophageal carcinoma](javascript:;) tumor tissues and normal tissues. (B) LINC00158 expression in [esophageal carcinoma](javascript:;) tumor tissues and normal tissues. (C) LINC00524 expression in [esophageal carcinoma](javascript:;) tumor tissues and normal tissues. (D) TPT1-AS1 expression in [esophageal carcinoma](javascript:;) tumor tissues and normal tissues.


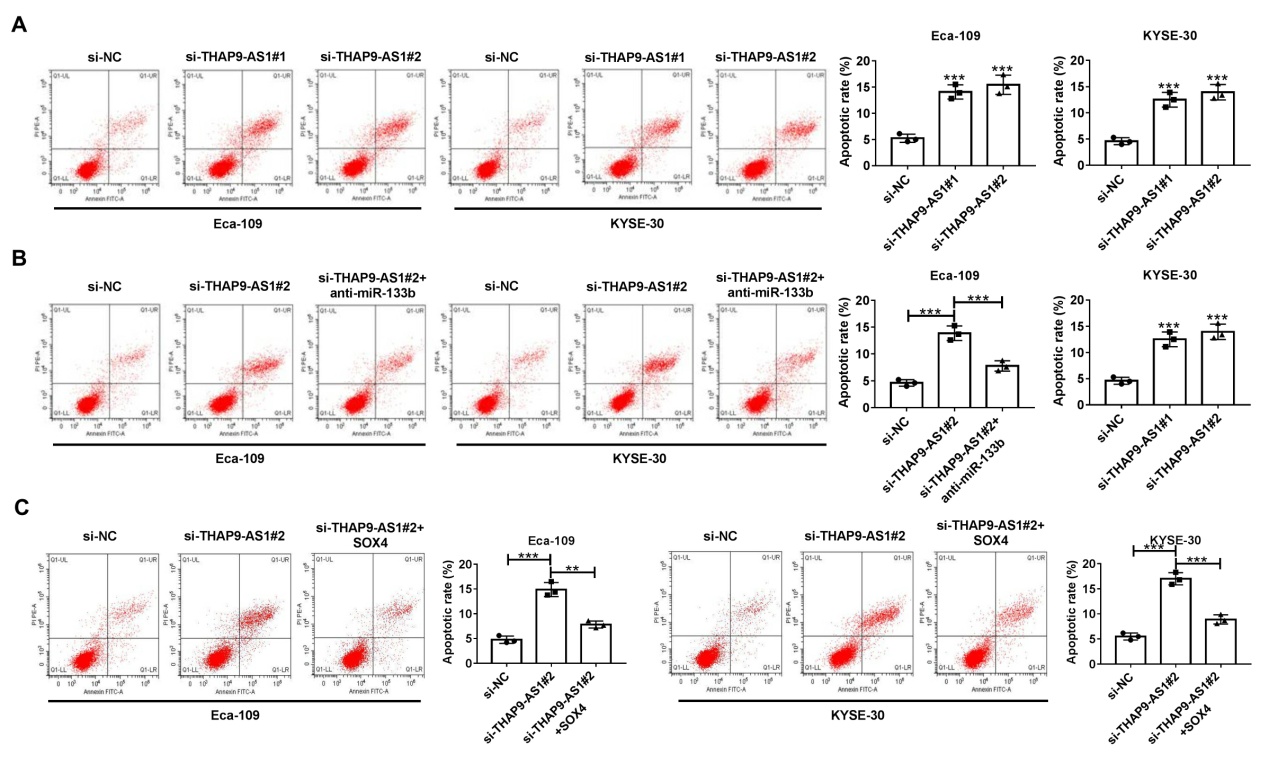


**Supplementary Figure S2.** (A) The apoptotic rate was analyzed by flow cytometry in Eca-109 and KYSE-30 cells with THAP9-AS1 knockdown. (B) Apoptosis was determined by flow cytometry in Eca-109 and KYSE-30 cells transfected with si-NC, si-THAP9-AS1#2 or si-THAP9-AS1#2+anti-miR-133b. (C) Flow cytometry was applied to assess the apoptosis of Eca-109 and KYSE-30 cells after transfection with si-NC, si-THAP9-AS1#2 or si-THAP9-AS1#2+SOX4.

**
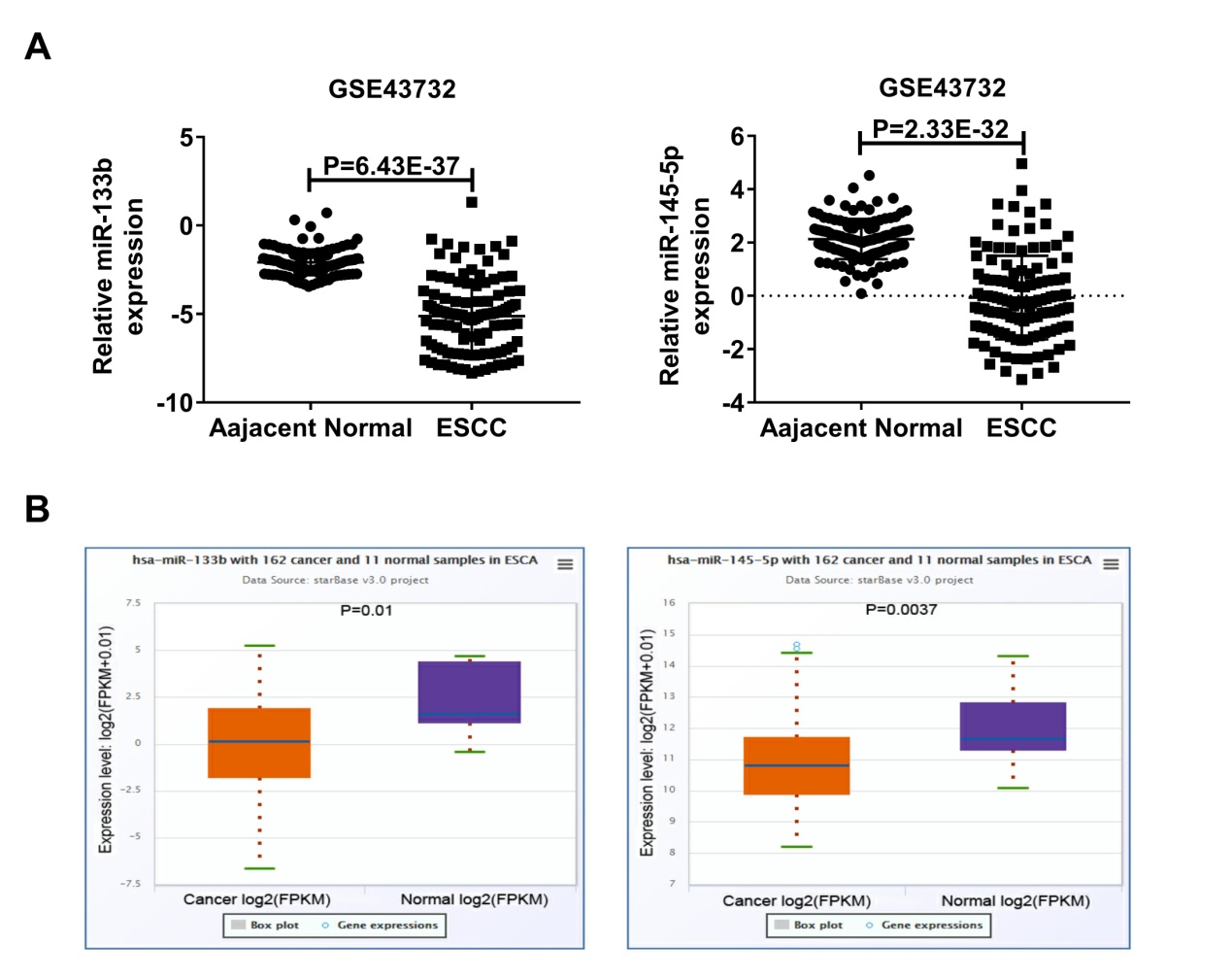
**

**Supplementary Figure S3.** The expression level of miR-133b and miR-145-5p was analyzed in ESCC tumor tissues according to TCGA data from GSE43732 (A) and starBase (B).


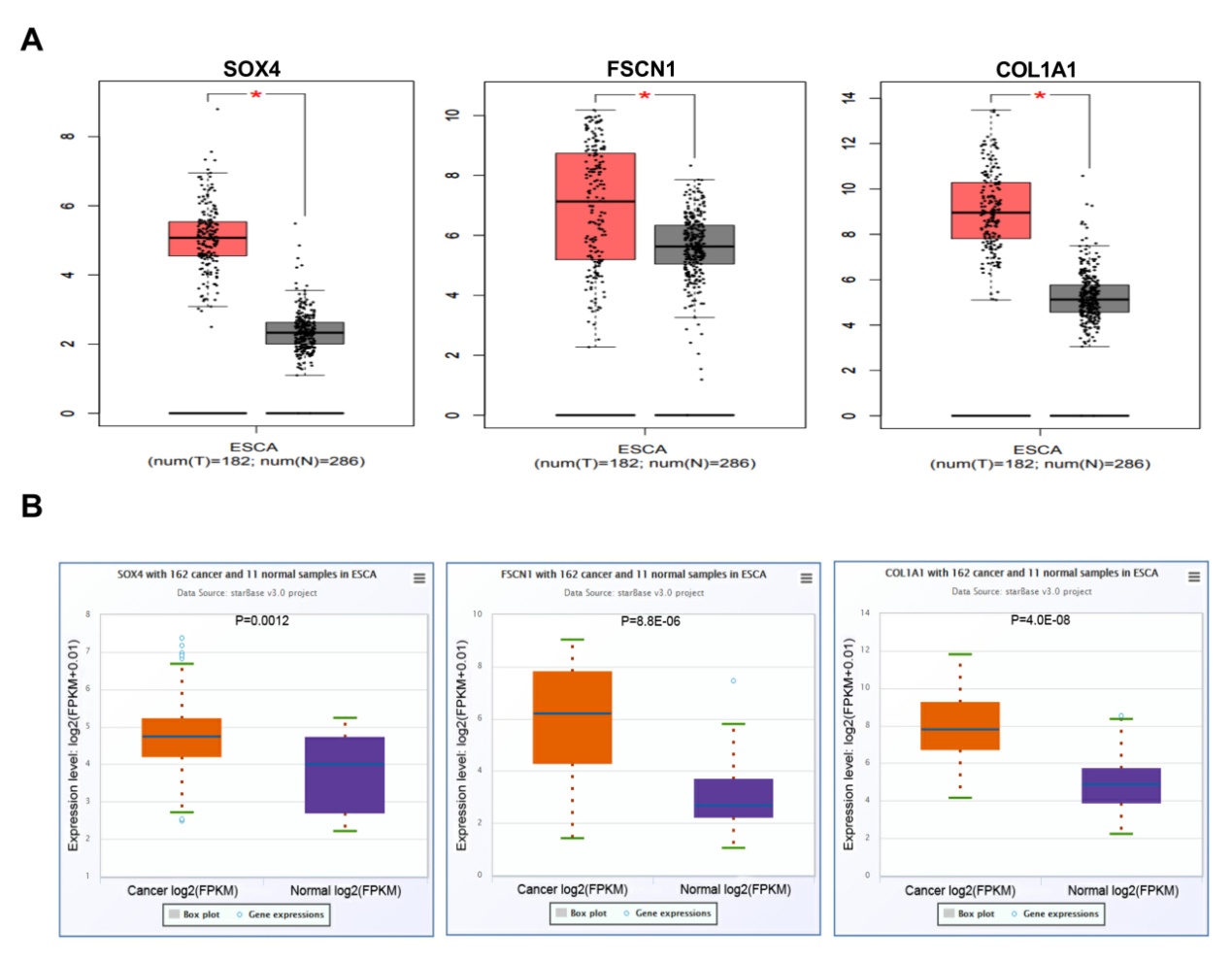


**Supplementary Figure S4.** GEPIA website (A) and starBase online tool (B) were used to analyze the expression difference of SOX4, FSCN1 and COL1A1 between [esophageal carcinoma](javascript:;) tissues and normal esophageal tissues.
