# Supplementary material for: THAP9-AS1/miR-133b/SOX4 positive feedback loop facilitates the progression of esophageal squamous cell carcinoma
Source: Cell Death Dis. 2021 Apr 14;12(4):401. doi: 10.1038/s41419-021-03690-z (PMC8046801; doi:10.1038/s41419-021-03690-z)
Supplement: Supplementary file 2 — Supplementary Tables [file 41419_2021_3690_MOESM2_ESM.docx]

**Supplementary Tables**

**Supplementary Table S1** Correlation between THAP9-AS1 expression and clinicopathological features of ESCC patients

| Variable | THAP9-AS1 expression | | *P* value |
| --- | --- | --- | --- |
|  | Low (n=34) | High (n=34) |  |
| Age |  |  | 0.618 |
| ≤ 60 | 20 | 22 |  |
| > 60 | 14 | 12 |  |
| Gender |  |  | 0.431 |
| Male | 22 | 25 |  |
| Female | 12 | 9 |  |
| Tumor size |  |  | 0.015* |
| < 5 cm | 20 | 10 |  |
| ≥ 5 cm | 14 | 24 |  |
| TNM stage |  |  | 0.013* |
| I/II | 18 | 8 |  |
| III/IV | 16 | 26 |  |
| Lymph node metastasis |  |  | 0.028* |
| Absent | 20 | 11 |  |
| Present | 14 | 23 |  |
| Differentiation grade |  |  | 0.300 |
| Well/moderate | 25 | 21 |  |
| Poor | 9 | 13 |  |
| Smoking status |  |  |  |
| Yes | 18 | 22 | 0.324 |
| No | 16 | 12 |  |
| Alcohol consumption |  |  | 0.604 |
| Yes | 10 | 12 |  |
| No | 24 | 22 |  |

**P* < 0.05

**Supplementary Table S2** qRT-PCR primer sequences

| **Gene** | **Primer sequence (5’-3’)** | |
| --- | --- | --- |
| THAP9-AS1 | Forward | CGATGCGGAGATAATGGGGA |
| THAP9-AS1 | Reverse | TCCTTCCCTGCATATTTTGAGTAA |
| miR-133b | Forward | TTGGTCCCCTTCAACCAGCTA |
| miR-133b | Reverse | CAGTGCGTGTCGTGGAGT |
| SOX4 | Forward | CCAGTTCTTGCACGCTGTTT |
| SOX4 | Reverse | TGTTGCAAGGTAGGAAGCCA |
| GAPDH | Forward | GCACCGTCAAGGCTGAGAAC |
| GAPDH | Reverse | ATGGTGGTGAAGACGCCAGT |
| U6 | Forward | CTCGCTTCGGCAGCACA |
| U6 | Reverse | AACGCTTCACGAATTTGCGT |
