# Supplementary figures and images for: THAP9-AS1/miR-133b/SOX4 positive feedback loop facilitates the progression of esophageal squamous cell carcinoma
Source: Cell Death Dis. 2021 Apr 14;12(4):401. doi: 10.1038/s41419-021-03690-z (PMC8046801; doi:10.1038/s41419-021-03690-z)

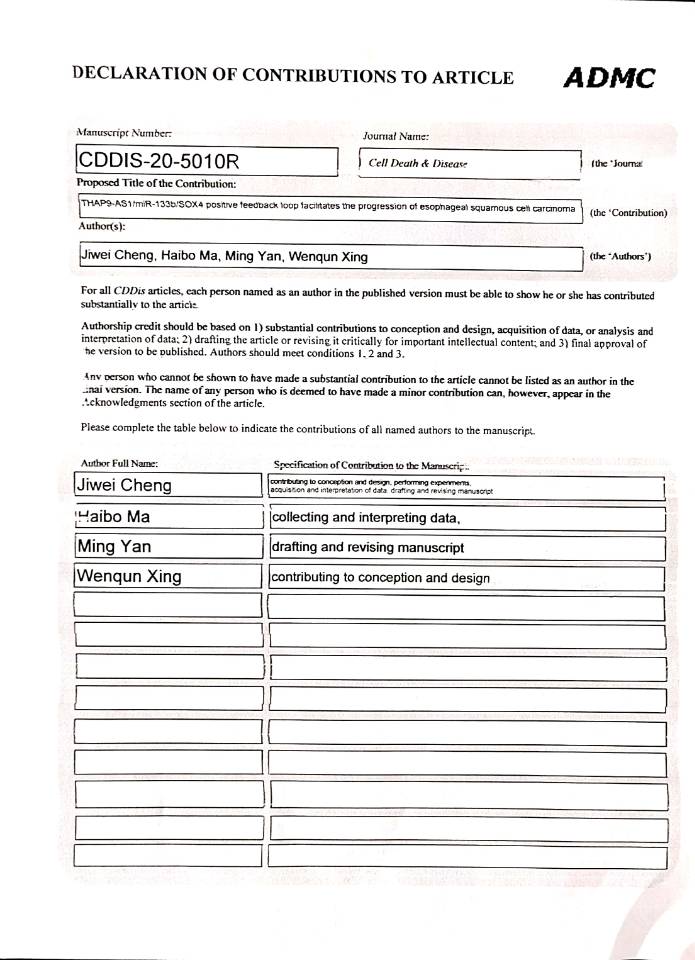


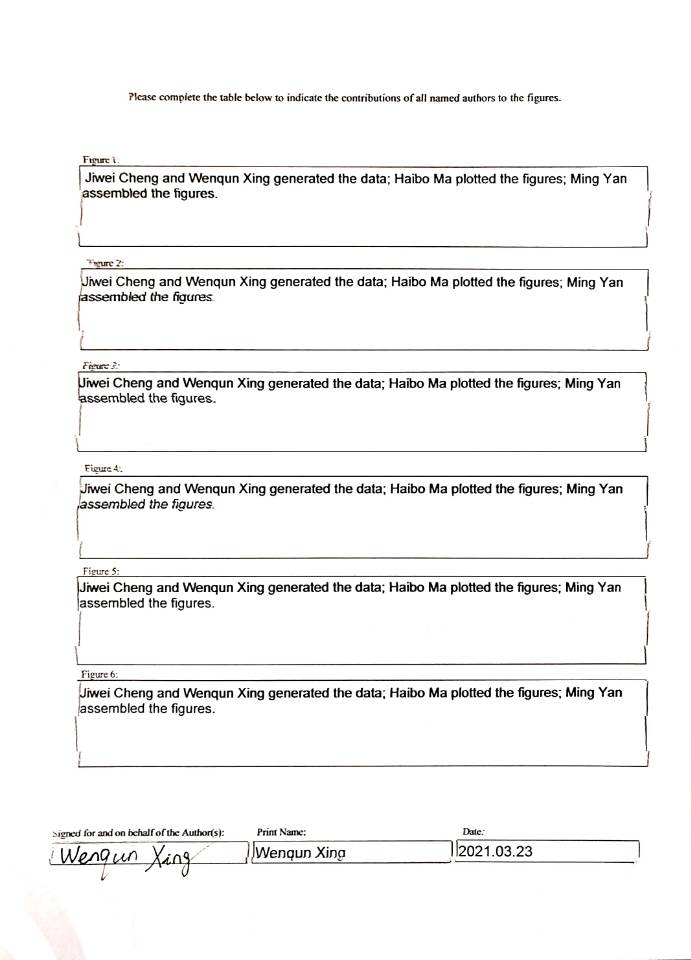

Supplement: Supplementary file 3 — Detailed Attribution of Authorship [file 41419_2021_3690_MOESM3_ESM.docx]

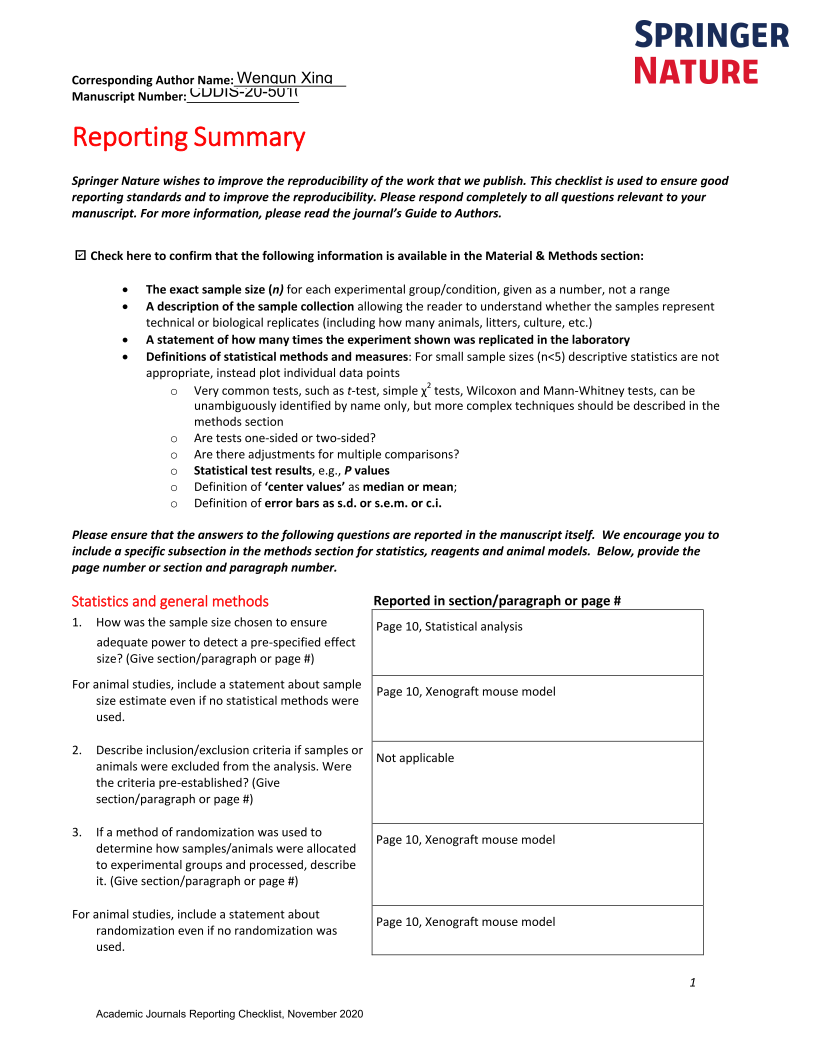


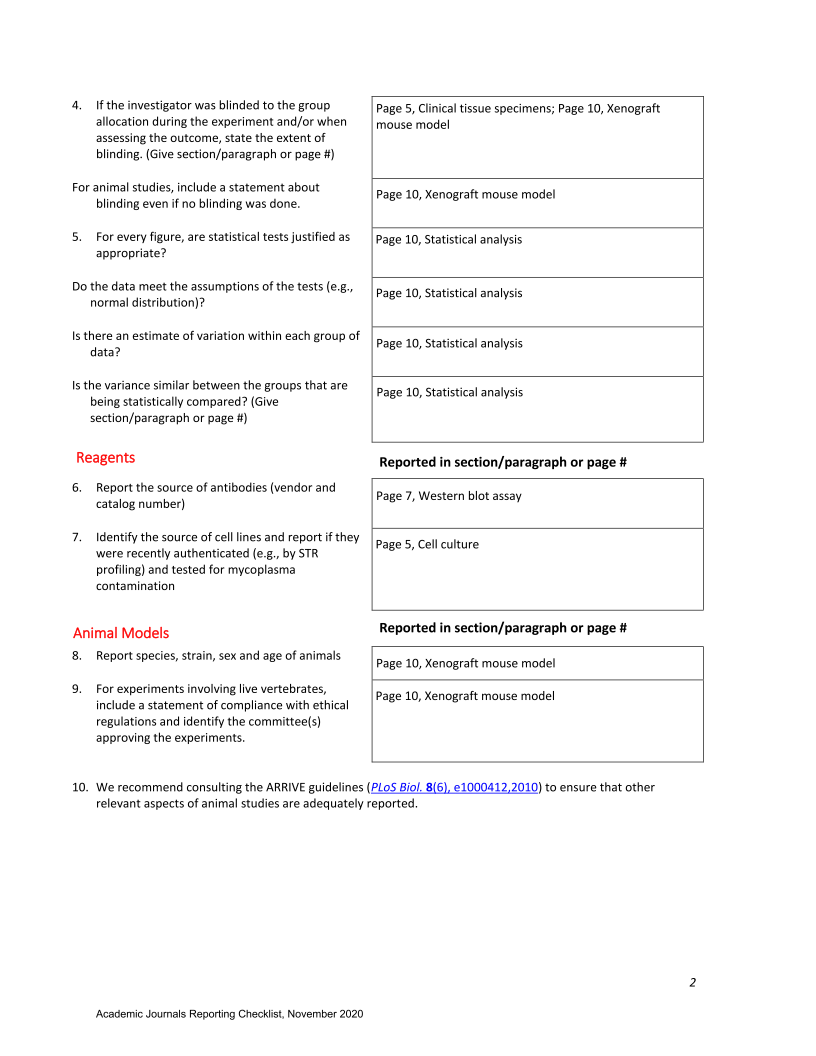


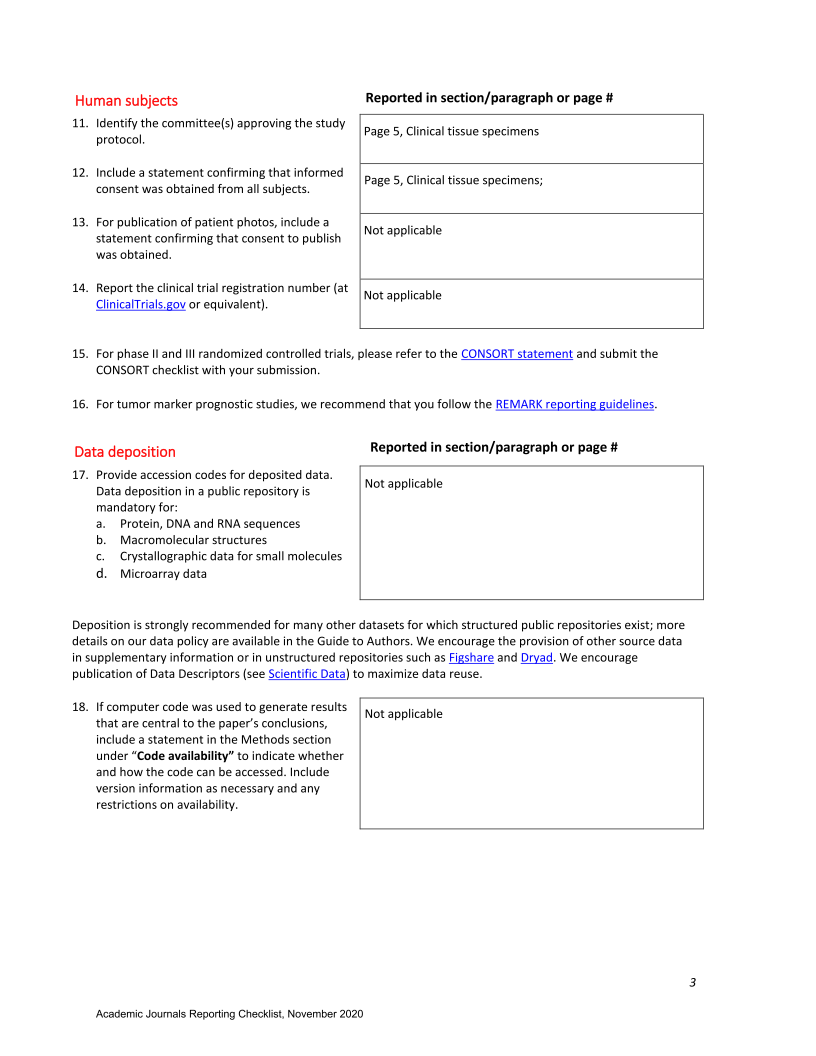

Supplement: Supplementary file 4 — Reproducibility Checklist Forms [file 41419_2021_3690_MOESM4_ESM.docx]
